# Effects of Spray-Drying Conditions on the Functional and Physicochemical Properties of Young Barley Grass Juice Powders

**DOI:** 10.3390/foods14101663

**Published:** 2025-05-08

**Authors:** Alicja Barańska-Dołomisiewicz, Joanna Żubernik, Katarzyna Samborska, Aleksandra Jedlińska, Dorota Witrowa-Rajchert

**Affiliations:** Department of Food Engineering and Process Management, Institute of Food Sciences, Warsaw University of Life Sciences (WULS-SGGW), 02-776 Warsaw, Poland; alicja_baranska@sggw.edu.pl (A.B.-D.); joannazubernik@hotmail.com (J.Ż.); katarzyna_samborska@sggw.edu.pl (K.S.); aleksandra_jedlinska@sggw.edu.pl (A.J.)

**Keywords:** spray-drying, young barley leaves, dehumidified air, antioxidant activity, bioactive compounds, retention coefficient, no carrier

## Abstract

Young barley leaves have been proven to distinguish themselves as highly potent in antioxidant activity, resulting from a high content of bioactive compounds. Due to their short storage time, it is crucial to prolong their shelf life. One of the methods that can be used is spray-drying, as it enables the production of powders that are highly valued in the food industry. This paper aimed to analyze the possibility of producing young barley leaf juice with improved properties. Juices were spray-dried with and without carriers at 100/60 °C inlet/outlet temperature using air of 1.5 g/m^3^ humidity as the drying medium. Maltodextrin (MD), Nutriose^®^ (N), and Arabic gum (AG) were used in a ratio 1:3 carrier/juice solids. The results proved that dehumidified air application enabled the production of young barley leaf juice powder, that was free of the carriers, of high retention coefficient (RC) of chlorophyll A and B (80.84 ± 6.56% and 87.05 ± 5.21%, respectively). No statistical difference was noted between variants with maltodextrin (chlorophyll A: 91.22 ± 5.07%, chlorophyll B: 71.72 ± 5.44%), Nutriose^®^ (chlorophyll A: 72.24 ± 5.32%, chlorophyll B: 67.04 ± 12.41%), and carrier-free powder; thus, the elimination of a carrier can be considered to effectively produce a “clean” label, functional product. The highest degradation among the tested bioactive compounds was noted for vitamin C.

## 1. Introduction

Enriching one’s diet with the young green parts of plants is a way to supplement and balance the nutritional value of the foods consumed on daily basis [1]. Fresh barley leaves are characterized by their high water content of up to 85–90%, which is a factor that causes them to have a relatively short shelf life after harvest. Unprocessed leaves undergo a number of physicochemical changes, leading to the loss of important nutrients or unfavorable color changes. Decreasing the water content in fresh leaves is an operation that allows their shelf like to be extended by lowering the risk of microbiological, chemical, and physical changes. Moreover, it provides new possibilities for their use as a stand-alone product or as an ingredient in many food formulations [2].

Paulíčková et al. (2007) studied young barley parts and they have proven their potential as a new, rich source of bioactive substances, with possible use in the food industry [3]. Moreover, there is a considerable amount of literature on the antioxidant potential of young barley. It has been shown that the young parts of barley are characterized by their strong ability to neutralize DPPH and ABTS radicals [4]. Comparative studies on the antioxidant potential of barley leaf extracts showed that compared to wheat leaf extracts, they display almost twice as high DPPH radical scavenging activity, as well as a higher ability to reduce and chelate iron ions, and also proved a higher content of polyphenols and phenolic acids [5]. It should be underlined that barley leaves are also a rich source of chlorophyll [6,7]. Chlorophyll content determinations in young barley juice, previously pasteurized and stored for a month, showed a significantly higher content compared to the other analyzed juices obtained from wheat and rice grasses [8]. Due to high content of bioactive compounds, young barley extracts may have an impact on minimizing the effects or have a preventive effect on many lifestyle diseases, including diabetes, circulatory system diseases, and joint inflammation, and also have a beneficial effect on stabilizing cholesterol levels and have anti-carcinogenic effects [9]. It has been found that they have a beneficial effect on the gastrointestinal tract and the neurological system, including potential properties that work against the occurrence of Alzheimer’s disease [4].

Spray-drying is a method used to remove water from liquid or semi-liquid feeds such as juices, extracts, pastes, or emulsions. This technique allows their shelf life to be extended and protects the compounds of health-beneficial properties, as powdered products are characterized by greater stability compared to the ingredients in liquid form [10]. There are alternative methods that allow for the production of powdered food from extracts, juices, and vegetable or fruit pastes, such as freeze-drying. However, spray-drying is the least energy-consuming and time-consuming method [11,12]. An undoubted advantage is the possibility of continuous production, which affects the efficiency of dryers [13]. Although the contact time of the material with the drying medium during spray-drying is short, it can lead to reduced biological activity and loss of valuable nutrients, as well as negatively affecting the taste, smell, or color of the product. Therefore, it is necessary to select optimal drying conditions for the selected material in order to minimize unfavorable changes during spray-drying [10].

A growing body of literature has investigated the application of dehumidified air as a drying medium in spray-drying in order to decrease the negative effects that might occur during the course of drying or to increase the quality of final product. Barańska et al. (2023) observed the easier course of drying while producing sour cherry juice concentrate powders, as the problem of stickiness decreased as a result of the application of dehumidified air [14]. Moreover, lowering the drying temperature, due to the use of air of lower humidity, enabled the production of sour cherry powders of 30% higher total phenolic content in comparison with powders that were spray-dried traditionally at a high drying temperature. Dehumidified air-assisted spray-drying is a method that makes it possible to reduce the carrier content or remove it completely. As a consequence of low drying temperatures, the risk of stickiness due to low glass transition temperature (Tg) decreases and it enables the production of pure, 100% powders. Samborska et al. (2021) obtained white mulberry molasses powders with only 10% content of a carrier [15]. In more recent studies, Samborska et al. (2024) produced powdered plant-based beverages with no additional carrier using conventional, high-temperature spray-drying; however, the authors noted low yield, thus they decided to apply dehumidified air as a drying medium in order to lower drying temperatures [16]. After the modification, they observed a successfully improved drying yield.

This paper aimed to analyze the possibility of producing young barley leaf juice powders with and without an additional carrier as a result of the application of dehumidified air as a drying medium in order to obtain powders of improved physiochemical properties. The tested carriers were maltodextrin, Arabic gum, and Nutriose^®^. Maltodextrin and Arabic gum were chosen due to their popularity in the food industry and research and their very good solubility in water solutions [17]. Nutriose^®^, characterized by its probiotic properties, was selected to add health-beneficial properties to final product [18]. The quality assessment of the obtained powders was performed by determining selected chemical properties: total phenolic content, chlorophyll A and B content, and vitamin C content, expressed as ascorbic acid content and antioxidant activity against the DPPH radical. Moreover, the physical properties were tested: dry matter content, water activity, hygroscopicity, particles morphology and size distribution, loose and tapped bulk densities, flowability, and color.

## 2. Materials and Methods

### 2.1. Materials

The raw materials used in the study were young barley leaves of the *Hordeum vulgare* variety *Sebastian* in Radzymin, Poland. The leaves used to obtain the juice came from crops grown in 2019–2020. The cultivation was carried out in an ecological way, without the use of chemical plant protection products. The leaves were harvested each time at a specific growth stage, when the height of the grass seedlings did not exceed 30 cm, and the green leaves had not entered the phase of transformation into ears. The leaves were mowed traditionally using a hand scythe. Immediately after cutting, the young barley leaves were stored for maximum of 7 days in special insulated polystyrene packaging at a maximum temperature of 5 °C.

### 2.2. Spray-Drying

To obtain juices for spray-drying from young barley leaves, a GSJ-620 slow juicer (Gotie, Zabrze, Poland) was used, operating at 45 rpm, using a fine-mesh sieve to prevent long grass fibers from getting into the juice. Freshly prepared juice was stored at 5 °C in a tight foil, string package wrapped in aluminum foil, without access to light and air for a maximum of 24 h and then subjected to a spray-drying process.

Young barley leaf juice was dried using a semi-technical MOBILE MINOR (GEA, Skanderborg, Denmark) spray-dryer. Dehumidified air was supplied to the spray-dryer as a drying medium using an air dehumidification system composed of a cooling unit TAEevo TECH020 (MTA, Codogno, Italy), a condensation unit (SWEGON, Gothenburg, Sweden), and an adsorption unit ML270 (MUNTERS, Kista, Sweden), reaching the air humidity of 1.5 g/m^3^. The juice was fed to the spray-dryer using a peristaltic pump, dosing the liquid feed at rate of 25 mL/min. The disk rotation speed was 24,000 rpm. The air temperatures at the inlet and outlet were 100 and 60 °C, respectively.

The liquid feeds of 50% solids concentration were prepared using three different carriers: maltodextrin (MD; MALTOSWEET 180 DE 17.0–19.9, Tate & Lyle, London, UK), Arabic gum (AG; Colian, Opatówek, Poland), and Nutriose (N; Roquette, Lestrem, France). The liquid feeds with the addition of a carrier were each prepared in a ratio 1:3 carrier/juice solids. This ratio was chosen based on the preliminary research that was tested to produce powders of the highest possible share of raw material in the powder; however, a carrier was included to verify their protective role in bioactive compound degradation and effect on the course of drying. Moreover, young barley leaf juice without the addition of carrier was spray-dried as well (NC), which was possible only as a consequence of the application of dehumidified air as a drying medium. The spray-drying was repeated twice for each variant.

### 2.3. Powder Characterization

#### 2.3.1. Dry Matter Content

The dry matter content (DM) was determined in accordance with the standard oven method. Approximately 1 g of sample was weighed and put in the dryer at 105 °C for 4 h.

#### 2.3.2. Water Activity

Water activity (*a_w_*) was measured using a HydroLab C1 hygrometer (Rotronic, Bassersdorf, Switzerland). The determination was made in constant conditions in a climatic chamber, where the air temperature was 25 °C.

#### 2.3.3. Hygroscopicity

The water vapor adsorption from the saturated NaCl solution was determined, ensuring the water activity of the environment at the level of 0.75. In order to examine the hygroscopic properties of the powders, the samples were weighed on an analytical balance with an accuracy of ±0.0001 g and placed in aluminum weighing vessels for 72 h in a desiccator at a controlled temperature of 25 °C. The change in the sample mass was verified and measurements were taken after 0.5, 1, 2, 4, 6, 24, 48, and 72 h. The obtained results were used to determine the kinetics of water vapor sorption as a change in the relative water content (U) during sorption based on the Fick’s second law [19]:

$$\frac{U_{r}-U}{U_{r}-U_{0}}=A\cdot exp(-K\cdot \tau )$$

where *U* is the relative water content, *U_r_* is the relative equilibrium water content, *U*_0_ is the relative initial water content, *A* is the constant parameter, *K* is the equation coefficient, and *τ* is the time [h].

The relative water content (*U*), relative equilibrium water content (*U_r_*), and relative initial water content (*U*_0_) were calculated by dividing the respective water content values (*u*, *u_r_*, *u*_0_, expressed in kg H_2_O/kg d.m.) by the initial water content, *u*_0_.

#### 2.3.4. Particle Morphology

In order to examine the particle morphology of powders, pictures were taken at 1500× magnification using a Hitachi TM3000 (Hitachi, Tokyo, Japan) scanning electron microscope (SEM) with an electron generator with an accelerating voltage of 20 kV. To prepare the samples for a measurement, powders were attached to the measuring table using carbon tape and placed in a Cressington 108auto coater (EO Elektronen-Optik-Service GmbH, Dortmund, Germany) to be covered with a layer of gold.

#### 2.3.5. Particle Size Distribution

The particle size distribution of powders was determined by laser diffraction using a CILAS 1190 particle size analyzer (Cilas SA, Orléans, France). The measurement was performed in wet dispersion in ethyl alcohol. The analysis of the dispersed particles was performed at 10% obscuration. The median particle diameter was expressed as *D*_50_.

#### 2.3.6. Loose and Tapped Bulk Densities

The loose bulk density (ρ_L_) was determined by weighing approximately 10 g of powder into a measuring cylinder with a volume of 25 mL. Then, the volume was read from the cylinder scale, with an accuracy of 0.5 mL.

The tapped bulk density (ρ_T_) was determined using a STAV 2003/Engelsmann AG volumeter (Engelsmann, Ludwigshafen, Germany). For this purpose, the sample prepared for determining the loose bulk density was used, which was then tapped 100 times. The volume occupied by the powder was read again, with an accuracy of 0.5 mL.

#### 2.3.7. Flowability

The flowability of produced powders was expressed as the Hausner ratio (*HR*) and calculated as a ratio of tapped bulk density (ρ_T_) to loose bulk density (ρ_L_).

#### 2.3.8. Color

The color parameters were measured using a Konica-Minolta CM-5 colorimeter (Konica-Minolta, Tokyo, Japan) in the CIE L*a*b* system, where L* indicated lightness, a* the share of red/green, and b* the share of yellow/blue. The following color parameters were calculated:

- Chroma (*C*) expressed as: $C=\sqrt{{\left(a^{*}\right)}^{2}+({b^{*})}^{2}}$;
- Hue (*h*) expressed as: $h={tan}^{-1}(\frac{b^{*}}{a^{*}})$.

### 2.4. Chemical Properties of Powders

#### 2.4.1. Total Phenolic Content

The total phenolic content (TPC) was determined using the spectrophotometric method, according to Nowacka et al. (2019), using the Folin–Ciocalteu reagent in fresh young barley leaf juice and powders [20]. A weighed amount of powder and juice was added into 50 mL glass beakers wrapped in aluminum foil to protect the samples against the destructive effects of light and then 25 mL of 80% ethanol was added. The sample weight of powder and juice was determined based on the dry matter content analysis, ensuring that the dry matter content in the juice and powder corresponded to the dry matter content of the samples prepared from fresh young barley leaves. The resulting mixture was homogenized using an IKA T10 homogenizer (IKA-Werke GmbH & Co. KG, Staufen im Breisgau, Germany) for 60 s at a blade rotation speed of 30 000 rpm. After homogenization, the blade was rinsed with 80% ethanol to ensure the complete transfer of the material into the beaker. The extract was then transferred onto a heating plate, where the beakers were covered and heated for 1 min after reaching boiling point. The extracts obtained were quantitatively transferred into 50 mL falcons, after prior filtration through filter paper. The volume was then filled up to 50 mL with 80% ethanol.

A measure of 0.15 mL of filtered extract was transferred to glass test tubes using pipettes; then, 4.2 mL of distilled water and 0.25 mL of Folin–Ciocalteu’s reagent were added. The mixture in the test tube was thoroughly mixed using Vortex and after 3 min, 0.5 mL of sodium carbonate solution with a concentration of 17.7% was added. The content was again thoroughly mixed using Vortex, placed in a dark place for 60 min. A blank sample was prepared, in which the volume of the extract was replaced with distilled water. After the incubation time, the absorbance was measured using a spectrophotometer (Heλios γ Thermo Spectronic, Waltham, MA, USA). The measurement was taken by zeroing the device against a blank sample at a length of 750 nm. The content of polyphenols was read from the prepared gallic acid standard curve, which was converted to mg of gallic acid in 100 g of solids.

#### 2.4.2. Vitamin C

Vitamin C content expressed as ascorbic acid was determined in young barley leaf juice and in powders. The analysis was performed according to the methodology presented by Spínola et al. (2012), using ultra-performance liquid chromatography (UPLC) [21].

The samples were extracted using the reagent prepared with distilled water with 3% addition of metaphosphoric acid and 8% addition of glacial acetic acid (99.5% concentration). The solutions were then shaken on a Vortex while protecting the falcons from sunlight with aluminum foil. The samples were centrifuged at 6000 rpm for 10 min at a thermostat setting of 4 °C. The extracts obtained in this way were decanted from the sediment for filtration through PTFE syringe filters with a pore diameter of 0.22 μm and poured into glass chromatographic vials, and then diluted 1:1 with the mobile phase and placed in the autosampler of the liquid chromatograph, in which the temperature was controlled at 4 °C. The mobile phase was a solution of 0.1% formic acid, prepared using the Millipore system (Millipore Direct-Q^®^ 3, Merck, Darmstadt, Germany). The extraction process was carried out three times.

Vitamin C content was analyzed in an Ultra Performance Liquid Chromatography (UPLC) using an ACQUITY device for determinations (Waters, Milford, CT, USA). The detection of compounds was carried out using a UV detector with a PDA photodiode array. Each time, the column was conditioned with the mobile phase used during the determinations. The chromatographic column used in the analysis was packed with HSS T3 silica bed, modified with hydrophobic C18 ligands. The temperature maintained in the chromatographic column oven was 25 °C. The eluent flow through the column was 0.25 mL/min. The analysis of the obtained chromatograms was performed at a wavelength of 245 nm, where the maximum of ascorbic acid peaks occurred. The results were presented in mg/100 g solids.

#### 2.4.3. Chlorophyll

The content of chlorophyll A and chlorophyll B were analyzed in young barley leaf juice and the produced powders using ultra-performance liquid chromatography (UPLC), according to the methodology presented by Guzman et al. (2012) [22].

Chlorophyll extraction was carried out according to the procedure presented by Śledź et al. (2016) [23]. Approximately 0.1 g of magnesium carbonate was added to each weighted sample, the purpose of which was to neutralize the acids present in the leaves, stabilize magnesium ions, and facilitate centrifugation and sedimentation. Centrifuge tubes with the samples were filled with an organic solvent (20 mL of 80% acetone solution). Then, the material was homogenized to intensify the extraction using an IKA laboratory homogenizer (D125 Basic, Sigma-Aldrich, Saint Louis, MS, USA) at a set speed of 30,000 rpm for 1 min. The next step was shaking using a Vortex test tube shaker at a set speed of 2400 rpm for 2 min. The samples were centrifuged in the MPW-260R centrifuge (Med. Instruments, Warsaw, Poland) for 6 min at 6000 rpm at 10 °C. The supernatant was poured off from the sediment and filtered through PTFE syringe filters with a pore size of 22 μm directly into glass chromatographic vials. The vials were placed in a liquid chromatograph autosampler, in which a constant temperature of 15 °C was maintained. The extraction processes were carried out three times.

The content of chlorophyll A and B was analyzed in an ACQUITY Ultra Performance Liquid Chromatography (UPLC) apparatus (Waters, Milford, CT, USA). The detection of compounds was performed using a UV detector with a PDA photodiode array. Each time, the column was conditioned with the mobile phase used during the determinations (eluent A—a mixture of acetonitrile, methanol and chloroform in the ratio 74/19/7; eluent B—0.05% ammonium acetate solution). A single injection of the extractant onto the chromatographic column (Waters HSS T3 C18), thermostated in an oven at 35 °C, was 10 μL. The flow rate of the mobile phase through the column was 0.25 mL/min. Empower 3 software (Waters, Milford, CT, USA) was used to record and analyze the spectra. Chromatograms obtained for chlorophyll A and B were analyzed at a wavelength of 650 nm. Identification was based on the retention time of individual chromatographic peaks and referred to reference substances. Results were presented in mg/g solids.

#### 2.4.4. Antioxidant Activity

The antioxidant activity was determined against the synthetic 1,1-diphenyl-2-picrylhydrazyl radical. The antioxidant activity was tested in young barley leaf juice and powders.

The concentrated solution of the DPPH radical was prepared by weighing 0.025 g of DPPH on an analytical balance (with an accuracy of ±0.0001 g). The weighed powder was transferred quantitatively to a 100 mL measuring flask, which was then filled with 99% methanol solution. The flask was protected from sunlight with aluminum foil. The obtained solution was mixed and then placed in cold temperature, and it was kept for no longer than 72 h. In order to prepare a working solution used for the determinations, 18 mL of the concentrated solution was taken, transferred to a measuring flask, and filled to 100 mL using 80% ethyl alcohol. Immediately before the assay, the absorbance of the working solution was checked at a wavelength of 515 nm using a Heλios γ Thermo Spectronic spectrophotometer (Waltham, MA, USA). The instrument was zeroed on an 80% aqueous solution of ethyl alcohol.

The control sample consisted of a 2 mL working solution of the DPPH radical with 2 mL of 80% ethyl alcohol solution and mixed until a homogeneous mixture was obtained. The control sample was stored in a dark place for 30 min, after which its absorbance was measured. To determine the antioxidant activity of young barley leaf juice and powders, extracts, 80% ethanol solution and DPPH working solution were mixed, stored in a dark place for 30 min, and the absorbance was measured at a wavelength of 515 nm.

The obtained results were the basis for determining the linear relationship between the efficiency of radical quenching and the concentration of the extract and determining the EC_50_ (efficient concentration). The EC_50_ parameter indicates the concentration of the extract necessary to neutralize 50% of free radicals.

#### 2.4.5. Retention Coefficient

The retention coefficient (RC) was calculated based on the method proposed by Samborska et al. (2021) [15]. The RC of bioactive compounds in the produced powders was presented as a percentage comparison of the values of selected bioactive compound in the raw material present in the powder with the raw material before drying, excluding the content of the carrier added.

### 2.5. Statistical Methods

The experiments were conducted in triplicate, and the results are expressed as the mean ± standard deviation. To assess significant differences between the mean values of the analyzed parameters, ANOVA and Tukey tests were carried out, with a significance level of α = 0.05.

## 3. Results and Discussion

### 3.1. Powder Characterization

#### 3.1.1. Dry Matter Content

The lowest dry matter content (*DM*) was observed for the powder spray-dried without the addition of a carrier (NC)—94.86 ± 0.04% (Table 1). In the case of other powders, *DM* was higher and was noted at a level of 95.98 ± 0.01% for Arabic gum (AG), 96.29 ± 0.04% for Nutriose^®^ (N), and 96.51 ± 0.26% for maltodextrin (MD) (Table 1). At the same time, no statistical difference in the dry matter content was shown between the samples obtained with the addition of a carrier, regardless of its type. An increase in the dry matter content due to the addition of carriers was also observed in kale leaves extracts while spray-drying [24]. It should be underlined that all of the produced powders were characterized by a water content no higher than 5%, which is considered a level that ensures safety during storage [25].

Similarly, the water content of spray-dried spinach juice without the addition of drying carriers was higher than in spinach powders with different variants of carriers. Spinach powder with the addition of Arabic gum was characterized by a higher water content than in the powder with maltodextrin [26]. This is the consequence of the difference in the structure of the carriers—Arabic gum compared to maltodextrin has branches containing shorter chains with more hydrophilic groups [27].

#### 3.1.2. Water Activity

All analyzed powders were characterized by water activity (*a_w_*) not exceeding 0.3 (Table 1). This signifies that the risk of microbiological growth and non-enzymatic browning processes, such as the Maillard reaction, is lowered. The *a_w_* of the powder with Nutriose^®^ (N) as a carrier was the lowest and significantly different from the *a_w_* of the powder without the addition of a carrier (NC). Despite the lack of significant differences, the powders with Arabic gum (AG) were characterized by the highest *a_w_* value compared to the values of the powders with other carriers (N and MD). This could be related to the hydrophilic structure of Arabic gum [28].

#### 3.1.3. Hygroscopicity

**Figure 1:**
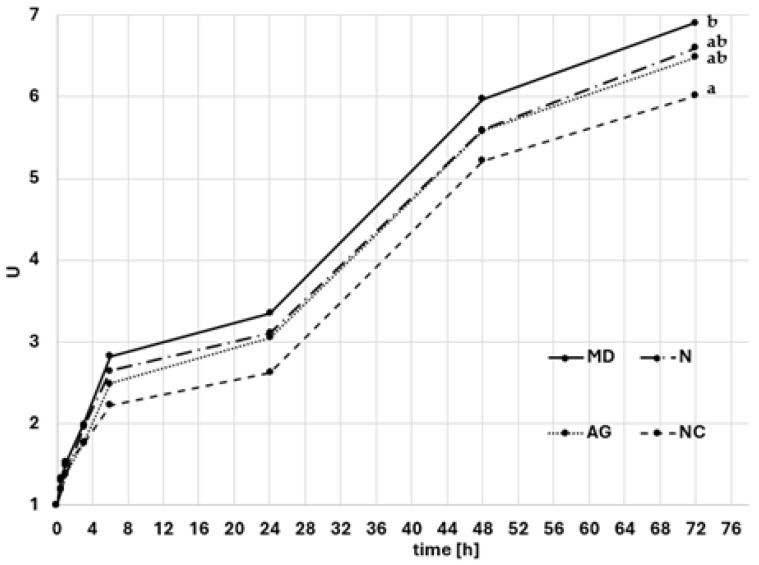
Water vapor sorption kinetics of spray-dried young barley leaf juices with maltodextrin (MD), Nutriose^®^ (N), or Arabic gum (AG) as carriers and with no additional carrier (NC) during humidification in an environment with *a_w_* = 0.75. ^a,b^—the same letters indicate homogeneous groups in statistical terms (Tukey’s test; *p*-value < 0.05). Tukey’s test was performed for the values collected after 72 h.

**Figure 2:**
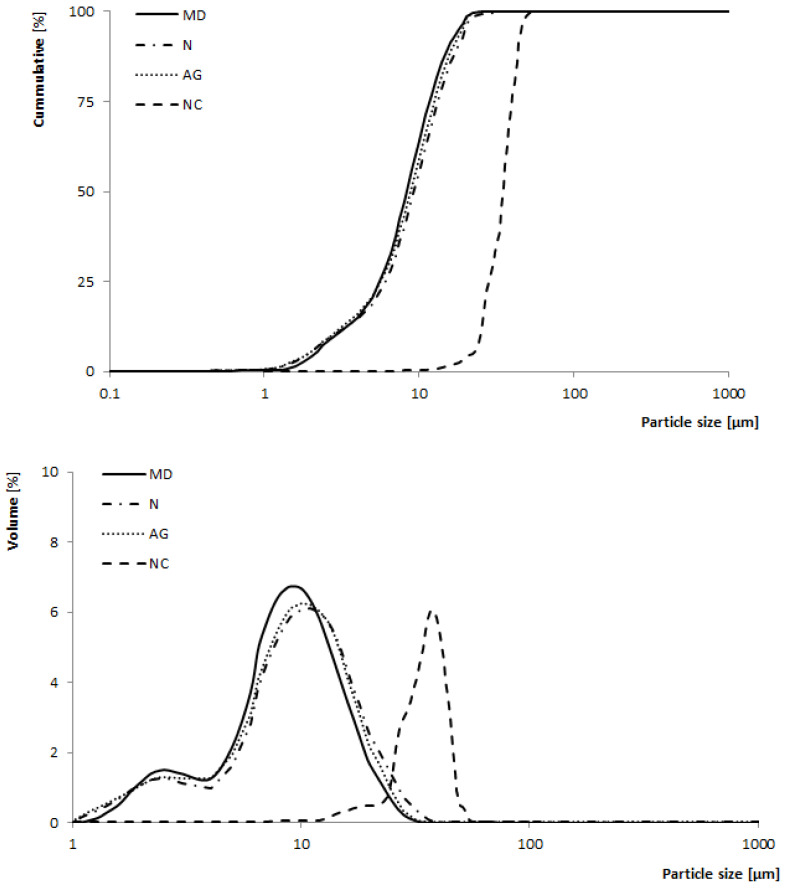
Cumulative particle size distribution and particle size distribution of young barley leaf juice powders with maltodextrin (MD), Nutriose^®^ (N), or Arabic gum (AG) as carriers and with no additional carrier (NC).

Among all the produced powders, the lowest water vapor adsorption capacity was demonstrated by a variant obtained without the additional carrier (NC; Figure 1), which was characterized by the largest particle size (Table 1), but at the same time had the smallest particle size distribution (Figure 2). Generally speaking, larger particles absorb less moisture from the environment as a consequence of smaller specific surface area in comparison to powders of smaller median particle diameter (*D*_50_); thus, the contact surface is smaller [29]. The obtained results in this research confirmed this relationship. This could have had an impact on the greater porosity (ε), which was indirectly confirmed by density measurements (the lowest loose and tapped bulk density) (Table 1). It should be noted that Jedlińska et al. (2021) observed the same relationship when spray-drying beetroot powders with the addition of different carriers and without a carrier [30]. The authors reported that powders produced with no additional carrier were characterized as having the lowest hygroscopicity.

The addition of carriers had an influence on the increased adsorption of moisture from the environment—the relative water content after 72 h of humidification increased from 7.8 to 15.0% compared to the powder obtained without a carrier (NC). A variant containing maltodextrin (MD) was characterized as having the greatest hygroscopicity—this powder increased its water content almost sevenfold during exposure for 72 h.

It has been demonstrated that carriers show a moisture sorption-inhibiting effect on powders, which is in contradiction with the results in this study. The authors, who studied the kuini powders, observed that increasing carrier content lowered the powders’ hygroscopicity [31]. Research on a spray-dried acai juice with three types of carriers indicated the significant importance of the chemical structure of the carriers and the raw material. The authors noted that the hygroscopicity of powders may result from the number of hydroxyl groups present in the powder. In the case of acai pulp dried using Arabic gum and maltodextrin, the powders obtained using Arabic gum showed lower water vapor adsorption [28]. On the other hand, the addition of Arabic gum to spray-dried pomegranate juice increased the hygroscopicity of the powder compared to the variant obtained with the addition of maltodextrin [32]. It is therefore not possible to unequivocally assess the influence of the type of carrier and presence of it, in general, on the water vapor adsorption capacity.

#### 3.1.4. Particle Size Distribution

The particle sizes of the produced powders were presented as the median particle diameter (*D*_50_, Table 1) and as cumulative particle size distribution and particle size distribution curves (Figure 2). The largest median particle diameter was observed for a powder obtained without carriers (NC; 11.61 ± 0.6 µm) (Table 1). It was the effect of increased stickiness, resulting from the lack of presence of a carrier. Generally speaking, greater *D*_50_ indicates a more problematic course of drying [14]. Jedlińska et al. (2021) observed a similar relationship when spray-drying beetroot powders with no additional carrier. Variants with added carriers were characterized as having smaller *D*_50_ [30]. The effect of carrier type on *D*_50_ was not noted as there were no differences between variants with different carriers.

Powders produced with the addition of carriers were characterized as having bimodal and wide particle size distribution curves. This type of particle size distribution curve may correspond to the efficiency of the filling of the spaces between large particles by smaller ones. The presence of particles of different sizes improves the packing of particles in the bed and increases the density, which was confirmed by higher densities noted for the variants with the addition of different carriers (Table 1).

#### 3.1.5. Particle Morphology

**Figure 3:**
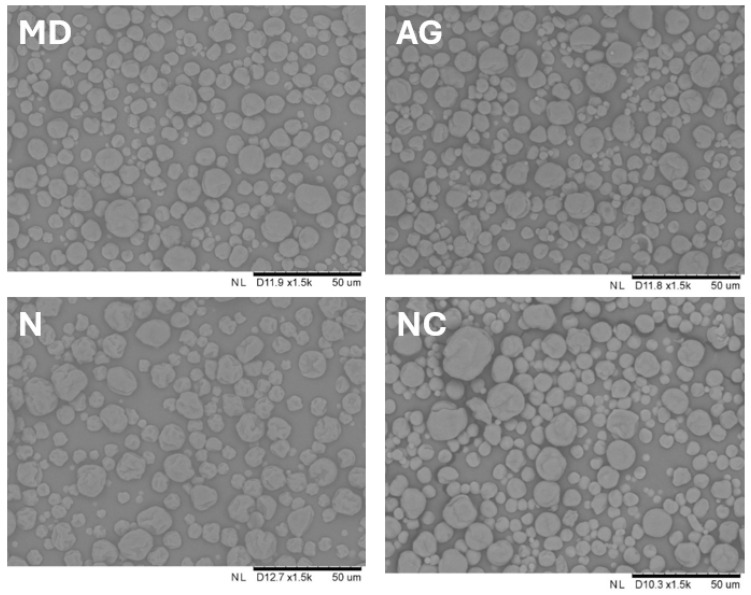
Scanning electron microphotographs (SEM) of young barley leaf juice powders with maltodextrin (MD), Arabic gum (AG), or Nutriose^®^ (N) as carriers and with no additional carrier (NC).

Analysis of scanning electron microscope images (SEM) (Figure 3) showed that all of obtained powders were characterized by an irregular structure of particles. There were visible dents and concavities on the surface of the walls. No broken particles were observed in any of the tested powders. The surface of powders with Arabic gum (AG) as a carrier seemed to be the most heterogeneous, with numerous irregularities, depressions, and wrinkles on the surface of the particles at the same time. A similar observation on the effect of the addition of Arabic gum on the differentiation of the structure of dried particles was noted for spray-dried blackberry juice, while the use of maltodextrin instead allowed particles with a smoother surface to be obtained and influenced the greater homogeneity of the obtained powders [33]. The phenomenon of irregular, wrinkled particle structure may be due to the use of relatively low inlet air temperature, which resulted in slower heat transfer to the material, and consequently slower, less intense evaporation, which led to the formation of irregularities on the surface of the formed particles [34].

#### 3.1.6. Loose and Tapped Bulk Densities

The powder obtained without the addition of a carrier (NC) was characterized by the lowest bulk density (*ρ_L_*, 486.38 ± 14.54 kg/m^3^; Table 1). This was due to the size of the particles of this powder being the largest. The density of powders decreases as the particle diameter increases. In that case, more free spaces filled with air are observed between large-sized particles, which results in occupying a larger volume and consequently having a lower bulk density [33].

The powders obtained with the addition of carriers (MD, N, AG) showed significantly higher bulk density values than the powders without the addition of carriers (NC). The highest *ρ_L_*, significantly different from the other variants of the analyzed powders, was noted for the powder obtained with the addition of maltodextrin (MD)—601.37 ± 9.08 kg/m^3^. At the same time, iMDwas the powder with the smallest particle size and the widest particle size distribution (Table 1, Figure 2).

Powders with the addition of carriers were characterized by a significantly higher tapped bulk density (*ρ_T_*) than the powders obtained without a carrier (NC; 628.89 ± 11.75 kg/m^3^; Table 1). The *ρ_T_* of young barley leaf juice powder obtained with Nutriose^®^ (N) and Arabic gum (AG) as carriers did not differ significantly.

Cao et al. (2017), who produced young barley grass powders with the addition of 20% of maltodextrin, observed similar values—the *ρ_L_* ranged from 640.26 ± 3.32 to 644.83 ± 3.32 kg/m^3^, while *ρ_T_* ranged from 774.28 ± 2.87 to 834.55 ± 3.12 kg/m^3^, depending on the drying temperature [35].

#### 3.1.7. Flowability

The powders’ flowability was expressed by the Hausner ratio (*HR*). An increase in the value of the *HR* indicates stronger intermolecular interactions and greater cohesion of powders [36,37]. The values of the HR below 1.25 indicate that the obtained powders can be classified as materials with good flowability. All obtained powders were characterized by a HR value below 1.25 (Table 1). The powder produced without carriers (NC) had the highest HR value (1.24 ± 0.02). The flowability of the powders with the carriers (AG, N, MD) did not differ from each other; thus, the effect of carrier type on this parameter was not observed.

#### 3.1.8. Color

**Figure 4:**
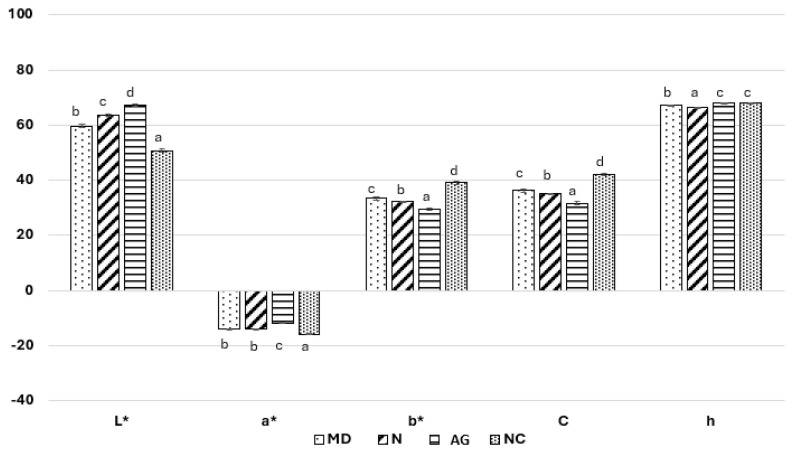
Color parameters (L*a*b*), chroma (*C*), and hue (*h*) of spray-dried young barley leaf juices. ^a–d^—the same letters indicate homogeneous groups in statistical terms (Tukey’s test; *p*-value < 0.05).

The powder obtained without carriers (NC) was characterized by the lowest lightness, significantly different from other powders (L* = 50.76 ± 0.63) (Figure 4). The variants obtained with the addition of carriers were lighter in color compared to the reference powder with no additional carrier (NC). The L* parameter of the powders with carriers ranged from 59.68 to 67.35, which increased in accordance with the order of the carriers used: maltodextrin < Nutriose^®^ < Arabic gum (MD < N < AG). According to Ferrari et al. (2012), the analysis of spray-dried blackberry powders showed significantly higher L* values of powder dried using maltodextrin compared to powder with Arabic gum [33]. The effect of the carrier content on the increase in the L* parameter value was also observed during the drying of young barley juice. It was noted that with the increase in the share of maltodextrin, the powder was lighter in color [35].

The powder obtained without the addition of carriers (NC) was characterized by the highest share of green color (a* = −15.82 ± 0.12) (Figure 4). It was the obvious effect of the highest share of juice in the powder. It should be underlined that the highest values of chlorophyll were noted for this variant, which justifies the obtained value for the a* parameter (Table 2). The largest decrease, over 25%, in the share of green color was noted in the case of the powder containing AG. As for the remaining powders produced with carriers, the decrease in the share of green color compared to the reference powder with no additional carrier (NC) was 11.6 and 10.6% containing N and MD, respectively; however, these variants were not statistically different from each other. In relation to b* parameter, the highest share of yellow was observed in powder produced without additional carriers (NC; b* = 39.10 ± 0.44) (Figure 3). There was a decrease in the share of yellow color by 25, 10, and 7%, respectively, in the case of powders with AG, N, and MD in comparison to the variant without a carrier (NC). The effect of carrier type and its addition was observed in the case of the b* color parameter.

Changes in the color parameters a* and b* are closely correlated with the color chroma (*C*), the values of which ranged from 31.69 ± 0.50 to 42.19 ± 0.46 (Figure 4). All powders spray-dried with the addition of carriers were characterized by a color of significantly lower *C* values than powder obtained without a carrier (NC), which resulted from a lower share of juice in the powder. At the same time, their color saturation was significantly different depending on the carrier type. The *C* values after the application of carrier decreased in the range of 14 to 28% compared to young barley leaf juice dried without a carrier (NC).

The hue (*h*) value of the powders ranged from 66.53 ± 0.06 to 68.16 ± 0.05 (Figure 4) and the influence of the carrier type was observed. The highest *h* value was noted for the powder obtained from juice spray-dried without additional carrier (NC) and with the use of Arabic gum (AG).

**Figure 5:**
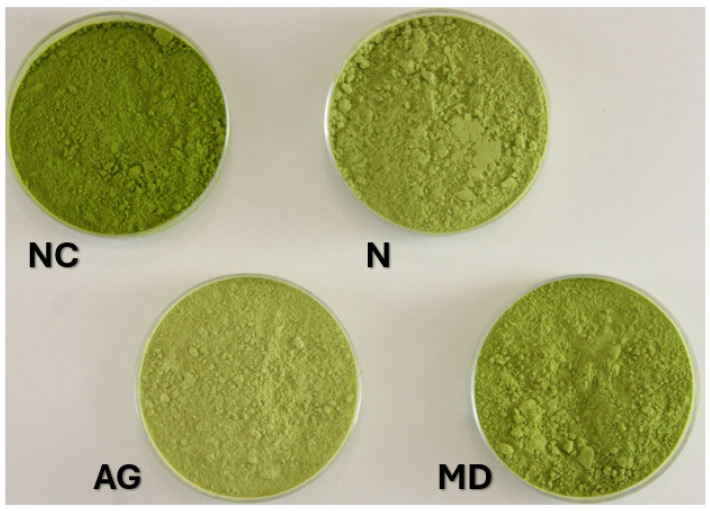
Pictures of young barley leaf juice powders without carrier (NC) and with the addition of Nutriose (N), gum Arabic (GA), and maltodextrin (MD).

To better illustrate the appearance of the powders, pictures were taken using a digital camera (Figure 5). It can be observed that, indeed, the powder without an additional carrier (NC) is notably distinguished from variants with carriers, which can also be confirmed with the human eye. It should be underlined that this was the effect of the highest share of juice in the powder, which was made possible by the application of dehumidified air.

### 3.2. Chemical Properties

#### 3.2.1. Total Phenolic Content

Raw juice from young barley leaves was characterized by a total phenolic content (TPC) of 2719 ± 34 mg/100 g solids (Table 2). Paulíčková et al. (2007) also observed a high TPC in barley juice—2804 mg/kg of sample after freezing and 15,793 mg/kg of sample after drying in a fluidized bed at 30 °C [3]. In comparison, juices from young rice leaves were characterized by a TPC of 31.44 ± 0.44 mg pyrogallol equivalent/g of extract and wheat grass by 33.7 4 ± 0.34 mg pyrogallol equivalent/g of extract [38]. All of resulting powders were characterized by a lower TPC than the value determined in the juice after pressing (Table 2). The highest TPC was observed in the powder obtained without the addition of carrier (NC) (1370 ± 20 mg/100 g solids), which was the obvious effect of the highest content of the raw material in the powder. This phenomenon was the consequence of dehumidified air application, which made it possible to remove the presence of a carrier in the powder completely.

The retention coefficient (RC) for the TPC of produced powders ranged from 25.70 ± 0.78 to 102.29 ± 5.14% (Table 2). It can be observed that the powder composition influenced the RC of the TPC. The highest retention was noted for the variant with maltodextrin as a carrier (MD). Akbarbaglu et al. (2021) and Michalska-Ciechanowska et al. (2021) reported that, depending on the type of a carrier, different relations to the protective degree of bioactive compounds during processing are presented [39,40]. It is worth highlighting that in the case of a variant with MD as a carrier, the retention exceeded 100%. The same phenomenon was observed by Chen et al. (2021) [41]. It is highly probable that in this case, new compounds were formed during the Maillard reaction, which reacted additionally with Folin reagent. The Folin–Ciocalteu method is known to be non-specific to assess total phenolic content, which might have led to falsely high results in this research. In the case of TPC, the results need to be interpreted with attention, as the F-C reagent can react with the compounds, which are not classified as phenolics and are naturally present in the raw material (such as ascorbic acid or reducing sugars) and can also appear as a result of the Maillard reaction during drying [42].

#### 3.2.2. Vitamin C

Ascorbic acid is a thermolabile compound that is also sensitive to light, oxygen, and the presence of some metals [43]. Losses of this component may occur during preliminary unit operations before the actual spray-drying process of the juice, such as crushing or cutting the raw material, which may lead to the activation of enzymes from the oxidase group, affecting the oxidation of L-ascorbic acid [44].

Freshly squeezed young barley leaf juice was characterized by an ascorbic acid content of 124.24 ± 12.63 mg/100 g solids. It should be underlined that this was a significant content of vitamin C in comparison with slowly squeezed juice from leaves of *Kalanchoe*, depending on the variety, which is characterized by an ascorbic acid content ranging from 9 to 81 mg/100 g solids [45]. Powder produced with Nutriose^®^ as a carrier (N) was distinguished by the lowest vitamin C content among all spray-dried powders (23.99 ± 2.14 mg/100 g solids) and it was 16% lower than that obtained in powder without a carrier (NC) (Table 2). The content of ascorbic acid in powders was not statistically different; however, taking into account the lower content of barley leaf juice in liquid feeds where some of the carriers were added (MD and N), the protective role of carriers needs to be highlighted in relation to powder without additional carriers.

The retention coefficient (RC) of vitamin C varied from 22.98 ± 1.21 to 28.70 ± 2.00% (Table 2) and a significant difference between variants was observed. The lowest retention was noted for the variant with no carrier addition (NC), which would signify that bioactive compound was not protected sufficiently. Thus, it can be concluded that when considering vitamin C content in young barley leaf juice powders, the presence of the carrier is essential. Large losses of ascorbic acid during spray-drying were observed by Rybak et al. (2020) for red pepper juice with maltodextrin [46]. Despite the low drying temperature as a consequence of the application of dehumidified air, degradation was noted at the level of 94% compared to the fresh sample. In the case of spray-dried spinach juice, a decrease in vitamin C content was also observed, which depended on the addition of the carrier and increased with increasing inlet and outlet temperatures of the drying medium. The addition of maltodextrin resulted in a 43% reduction in the content of ascorbic acid in the powder obtained from spinach juice, and the addition of Arabic gum resulted in a 22% reduction in the content compared to the powder without the addition of carriers. The authors indicated that the lower content of bioactive compounds in spray-dried powders obtained using carriers was the effect of lower share of raw material in the final product [26].

#### 3.2.3. Chlorophyll

In all of the powders produced with carriers, the content of chlorophyll A decreased significantly compared to the juice, in which the content was 7.01 ± 0.31 mg/g solids (J) (Table 2). Powders from young barley leaves differed with respect to the content of chlorophyll A. The highest content of chlorophyll A was observed in the powder obtained without the use of carriers (NC; 5.67 ± 0.46 mg/g solids). Powders that were spray-dried with carriers were characterized by a significantly lower content of chlorophyll A, which was the obvious effect of the composition of spray-dried variants, hence the chosen ratio of juice to carrier solids. The lowest content among powders with carriers was determined in the variant with Arabic gum (AG) and the highest was in the powder with maltodextrin (MD).

The retention coefficient (RC) of chlorophyll A ranged from 54.62 ± 4.97 to 91.22 ± 5.07% (Table 2). The effect of the presence of a carrier and its type on the chlorophyll A content was observed. The highest value was noted for the variant with maltodextrin (MD); thus, the protective effect of this type of a carrier was noted. However, as there was no significant difference between the powder with no additional carrier, it can be concluded that the elimination of a carrier can be considered in order to produce a “clean-label” product of very satisfactory retention.

A similar relationship was also noted for chlorophyll B, as it decreased after spray-drying in comparison to raw juice (Table 2). The powders differed significantly with respect to the content of chlorophyll B—the variant without carrier (NC) contained more chlorophyll B than the other variants, which was the effect of the highest share of raw material in final product.

The retention coefficient (RC) of chlorophyll B varied from 46.95 ± 4.96 to 87.05 ± 5.21% (Table 2) and, as in the case of chlorophyll A, the similar effect of carrier presence and type was noted, pointing to the favorable result of carrier elimination.

The application of carriers was chosen in order to verify the need to protect the bioactive compounds from potential degradation due to elevated temperature and to justify their use in comparison to powder without an added carrier. The obtained results suggest that Arabic gum (AG) played the least protective role towards chlorophyll A and B, contrary to its effect on the TPC and antioxidant activity. Maltodextrin and Nutriose^®^ (MD and N), however, turned out to have better protective properties in relation to both chlorophylls. Koç and Dirim (2017) also observed higher chlorophyll retention in spray-dried spinach leaf juice when using maltodextrin, in comparison to Arabic gum [43]. The results in this study are in accordance with the data presented in the research by Cao et al. (2017) [35]. The authors showed that with the increase in the maltodextrin content and the inlet air temperature, the chlorophyll content in the powders of spray-dried barley seedling extracts decreased. The highest chlorophyll content (7.29 ± 0.27 mg/g solids) was noted for the powder with 10% maltodextrin content and at the inlet temperature of 140 °C. A similar relationship was also observed for the spray-dried extract of moringa leaves with an increasing share of maltodextrin [47]. The addition of carriers also led to a significant decrease in the chlorophyll content in spray-dried spinach leaf juice. Koç and Dirim (2017) observed that powder without the addition of carriers characterized with the content of chlorophyll on the level of 347.93 ± 0.37 mg/kg solids and the addition of Arabic gum and maltodextrin caused a reduction in this value by 77 and 92%, respectively, in relation to the powder with no carrier [43].

#### 3.2.4. Antioxidant Activity

The concentration of extracts from young barley powders necessary to quench half of the initial amount of radicals, EC_50_, was determined—the lower the concentration achieved, the stronger the antioxidant effect of the extract.

The juice obtained after squeezing was characterized by the ability to neutralize free DPPH radicals at a level of 0.31 ± 0.06 mg solids/mL (Table 2). Spray-drying led to a decrease in antioxidant activity in all of the produced powders. The highest ability to quench the DPPH radical was noted for the powder dried without carriers (NC; DPPH = 0.50 ± 0.09 mg solids/mL) and with the addition of Arabic gum (AG; DPPH = 0.52 ± 0.05 mg solids/mL). As in the case of total phenolic content (TPC), this result confirms the protective role of Arabic gum as a carrier and, taking into account the reduced juice content in the variants containing carriers, it should be underlined that the remaining carriers (MD and N) also demonstrated a protective role towards bioactive compounds with antioxidant properties. However, powders obtained using MD and N as carriers were characterized by statistically significantly lower antioxidant activity (DPPH = 0.87 ± 0.06 and DPPH = 0.87 ± 0.06 mg solids/mL, respectively).

It can be concluded that Nutriose^®^ (N) as a carrier in the spray-drying of young barley juice resulted in the obtainment of powders with the lowest TPC and antioxidant activity against DPPH. Nutriose^®^, due to its physical and chemical properties such as low hygroscopicity, high solubility in aqueous solutions, resistance to high temperatures, and stability in a low-pH environment, has been used as one of the carriers in spray-drying. In addition, the added value resulting from the nutritional and prebiotic properties makes N an alternative to MD or AG [18].

## 4. Conclusions

1. The elimination of carriers in young barley leaf juice powders was possible, as a consequence of lowering the drying temperature as an effect of air dehumidification. This method enabled the obtainment of free-flowing carrier-free powder of satisfactory physiochemical properties, including a high retention coefficient (RC) of analyzed bioactive compounds, making it a “clean-label” product.
2. In relation to fresh juice, it should be underlined that low-temperature spray-drying caused the highest degradation of a vitamin C in comparison to other bioactive compounds, pointing to the highest thermolability of all tested compounds. Moreover, the protective role of different carriers in the spray-drying of young barley leaf juice needs to be addressed. There was no statistical difference observed between variants with maltodextrin and Nutriose^®^ and the carrier-free powder, considering lower share of raw material in liquid feeds where the carrier was present. Thus, spray-drying without a carrier can be considered as an approach to produce young barley leaf juice powder.
3. Dehumidified air-assisted spray-drying enabled powders with a retention of chlorophyll A and B exceeding 80% to be obtained when choosing proper drying conditions—as well as accounting for the carrier type and its presence—making it a method suitable for production of functional powders from very potent young barley grass juice. Similarly to the vitamin C retention coefficient, no statistical difference was noted between variants with maltodextrin and Nutriose^®^ and the carrier-free powder. Therefore, similar conclusions regarding the presence of a carrier can be drawn. However, as the retention coefficients of both chlorophylls are very satisfactory, the elimination of a carrier can be considered in order to obtain a functional product from young barley leaves that can be labeled as “clean”.
4. In order to achieve the most satisfying physiochemical properties of the final product when using a carrier, it is recommended to use Arabic gum (AG) as the most suitable carrier for the spray-drying of young barley leaf juice. This carrier showed the lowest hygroscopicity and the most homogenous particle morphology when added to young barley leaf juice.
5. In the case of the potential application of the produced powders as food colorants, it is recommended to use powders with no additional carrier or with the addition of maltodextrin or Nutriose^®^, as they were characterized by the greatest share of green color and color chroma.

## Figures and Tables

**Table 1:** Dry matter content (*DM*), water activity (*a_w_*), median particles diameter (*D*_50_), loose bulk density (*ρ_L_*), tapped bulk density (ρ_T_), flowability (*HR*) of produced powders with no carrier added (NC) and with maltodextrin (MD), Nutriose^®^(N), or Arabic gum (AG) as carriers.

	NC	MD	N	AG
*DM* [%]	94.86 ± 0.04 ^a^	96.51 ± 0.26 ^b^	96.29 ± 0.04 ^ab^	95.98 ± 0.01 ^ab^
*a_w_* [-]	0.27 ± 0.02 ^b^	0.18 ± 0.01 ^ab^	0.15 ± 0.01 ^a^	0.20 ± 0.01 ^ab^
*D*_50_ [µm]	11.61 ± 0.65 ^b^	8.77 ± 0.57 ^a^	9.41 ± 0.41 ^a^	8.89 ± 0.30 ^a^
*ρ_L_* [kg/m^3^]	486.38 ± 14.54 ^a^	601.37 ± 9.08 ^c^	549.42 ± 9.97 ^b^	565.07 ± 17.10 ^b^
*ρ_T_* [kg/m^3^]	628.89 ± 11.75 ^a^	715.57 ± 13.94 ^c^	670.70 ± 10.06 ^b^	658.58 ± 4.18 ^b^
*HR* [-]	1.24 ± 0.02 ^b^	1.19 ± 0.01 ^a^	1.20 ± 0.03 ^a^	1.20 ± 0.01 ^a^

^a–c^—the same letters indicate homogeneous groups in statistical terms (Tukey’s test; α = 0.05).

**Table 2:** Total phenolic content (TPC), vitamin C content, chlorophyll A and B content, and the antioxidant activity (EC_50_) of young barley leaf juice and powders with no carrier added (NC) and with maltodextrin (MD), Nutriose^®^ (N), or Arabic gum (AG) as carriers; retention coefficient (RC) calculated for bioactive compounds of produced powders.

	Juice	NC	MD	N	AG
TPC [mg GAE/100 g solids]	2719.19 ± 33.98 ^e^	1370.35 ± 19.76 ^d^	695.35 ± 34.96 ^b^	524.05 ± 15.81 ^a^	990.68 ± 47.11 ^c^
RC [%]	-	50.40 ± 0.73 ^b^	102.29 ± 5.14 ^c^	25.70 ± 0.78 ^a^	48.58 ± 2.31 ^b^
Vit. C [mg/100 g solids]	124.24 ± 12.63 ^b^	28.55 ± 1.50 ^a^	25.67 ± 1.94 ^a^	23.99 ± 2.14 ^a^	26.74 ± 1.86 ^a^
RC [%]	-	22.98 ± 1.21 ^a^	27.55 ± 2.08 ^ab^	25.75 ± 2.30 ^ab^	28.70 ± 2.00 ^b^
Chlorophyll A [mg/g solids]	7.01 ± 0.31 ^e^	5.67 ± 0.46 ^d^	4.80 ± 0.27 ^c^	3.80 ± 0.28 ^b^	2.87 ± 0.26 ^a^
RC [%]	-	80.84 ± 6.56 ^bc^	91.22 ± 5.07 ^c^	72.24 ± 5.32 ^b^	54.62 ± 4.97 ^a^
Chlorophyll B [mg/g solids]	2.83 ± 0.14 ^c^	2.47 ± 0.15 ^c^	1.52 ± 0.12 ^b^	1.42 ± 0.26 ^ab^	1.00 ± 0.11 ^a^
RC [%]	-	87.05 ± 5.21 ^b^	71.72 ± 5.44 ^b^	67.04 ± 12.41 ^b^	46.95 ± 4.96 ^a^
EC_50_ [mg solids/mL]	0.31 ± 0.06 ^a^	0.50 ± 0.09 ^b^	0.87 ± 0.06 ^c^	1.15 ± 0.08 ^d^	0.52 ± 0.05 ^b^

^a–e^ the same letters indicate homogeneous groups in statistical terms (Tukey’s test; *p*-value < 0.05).

## Data Availability

The original contributions presented in this study are included in the article. Further inquiries can be directed to the corresponding author.
